# Phospholipid Profiles for Phenotypic Characterization of Adipose-Derived Multipotent Mesenchymal Stromal Cells

**DOI:** 10.3389/fcell.2021.784405

**Published:** 2021-12-01

**Authors:** Janina Burk, Michaela Melzer, Alina Hagen, Katrin Susanne Lips, Katja Trinkaus, Ariane Nimptsch, Jenny Leopold

**Affiliations:** ^1^ Equine Clinic (Surgery, Orthopedics), Justus-Liebig-University of Giessen, Giessen, Germany; ^2^ Experimental Trauma Surgery, Faculty of Medicine, Justus-Liebig-University of Giessen, Giessen, Germany; ^3^ Institute for Medical Physics and Biophysics, Faculty of Medicine, University of Leipzig, Leipzig, Germany

**Keywords:** mesenchymal stromal cells, phenotyping, lipidomics, lipid markers, human, equine, platelet lysate, phosphatidylglycerol

## Abstract

Multipotent mesenchymal stromal cells (MSC) have emerged as therapeutic tools for a wide range of pathological conditions. Yet, the still existing deficits regarding MSC phenotype characterization and the resulting heterogeneity of MSC used in different preclinical and clinical studies hamper the translational success. In search for novel MSC characterization approaches to complement the traditional trilineage differentiation and immunophenotyping assays reliably across species and culture conditions, this study explored the applicability of lipid phenotyping for MSC characterization and discrimination. Human peripheral blood mononuclear cells (PBMC), human fibroblasts, and human and equine adipose-derived MSC were used to compare different mesodermal cell types and MSC from different species. For MSC, cells cultured in different conditions, including medium supplementation with either fetal bovine serum or platelet lysate as well as culture on collagen-coated dishes, were additionally investigated. After cell harvest, lipids were extracted by chloroform/methanol according to Bligh and Dyer. The lipid profiles were analysed by an untargeted approach using liquid chromatography coupled to mass spectrometry (LC-MS) with a reversed phase column and an ion trap mass spectrometer. In all samples, phospholipids and sphingomyelins were found, while other lipids were not detected with the current approach. The phospholipids included different species of phosphatidylcholine (PC), phosphatidylethanolamine (PE), phosphatidylinositol (PI) and phosphatidylserine (PS) in all cell types, whereas phosphatidylglycerol (PG) species were only present in MSC. MSC from both species showed a higher phospholipid species diversity than PBMC and fibroblasts. Few differences were found between MSC from different culture conditions, except that human MSC cultured with platelet lysate exhibited a unique phenotype in that they exclusively featured PE O-40:4, PG 38:6 and PG 40:6. In search for specific and inclusive candidate MSC lipid markers, we identified PE O-36:3 and PG 40:7 as potentially suitable markers across culture conditions, at which PE O-36:3 might even be used across species. On that basis, phospholipid phenotyping is a highly promising approach for MSC characterization, which might condone some heterogeneity within the MSC while still achieving a clear discrimination even from fibroblasts. Particularly the presence or absence of PG might emerge as a decisive criterion for future MSC characterization.

## Introduction

Multipotent mesenchymal stromal cells (MSC) are being explored as a therapeutic agent for a wide range of pathological conditions, in human as well as in veterinary medicine. While progress is evident, there is still a tremendous deficit regarding the characterization, definition and discrimination of this cell type. On the one hand, this results from the versatility of MSC characteristics depending on the MSC origin tissue (Hass et al., 2011; Burk et al., 2013; Fabre et al., 2019) and species (Schwarz et al., 2012; Hillmann et al., 2016), as well as on the culture conditions. Variations in the latter include culture media and supplements used (Schubert et al., 2018; Czapla et al., 2019; Hagen et al., 2021), 2D vs 3D culture and possible scaffold materials (Rashedi et al., 2017; Yang et al., 2018), or static vs dynamic culture and oxygen tension (Frith et al., 2010; Yeatts et al., 2013). However, on the other hand, MSC characterization is also impeded because current methodological approaches are afflicted with shortcomings.

Plastic-adherence, trilineage differentiation potential and the expression of certain surface antigens while lacking surface antigens of other cell lineages, as previously defined by the International Society for Cell and Gene Therapy (ISCT; Dominici et al., 2006), are still the only criteria which are mostly assessed in an acceptably standardized manner. However, the relevance and specificity of these characterization criteria are a matter of debates. Therefore, the ISCT has also recommended to complement this traditional characterization with functional assays (Galipeau et al., 2016), the choice of which depends on the therapeutic target and aim, thus this cannot be universally applied.

Moreover, the immunophenotyping approach is afflicted with methodological difficulties when it comes to the comparative characterization of cells from different species, which is crucial in the context of translational animal studies (Burk et al., 2018). In particular, the lack of direct comparability of immunophenotyping results, based on the different affinity of the antibodies used to identify specific antigens in different species, will always remain a challenge. As a consequence, it is currently still unclear if differences in the immunophenotype observed between MSC from different species are real or due to methodological issues. Hence, phenotyping approaches that are better suited to identify molecules and their composition across species would be advantageous and should complement traditional immunophenotyping.

Lipidomics are gaining growing attention and represent a promising novel approach for cell characterization (Meissen et al., 2012; Atilla-Gokcumen et al., 2014; Symons et al., 2021). Due to the implication of lipids in cell signaling, it seems promising that lipid profiles indicative of cellular potency can be identified (Meissen et al., 2012). A recent and comprehensive study demonstrated that different cell lineages have distinct lipidomic profiles, which was evident in whole membrane as well as in plasma membrane analyses (Symons et al., 2021). For human MSC derived from bone marrow, this study revealed that their plasma membranes contained high proportions of fully saturated and polyunsaturated fatty acids but less di-unsaturated lipids, and nearly no phosphatidylserine headgroups, which appeared to be distinct from other cell types. However, this previous study did not focus on MSC but mainly investigated different established cell lines, such as CHO or HEK cells (Symons et al., 2021), and studies focusing on MSC lipid patterns are still rare. So far, these studies addressed the differences and changes in lipid composition in young vs old donor MSC and during prolonged passaging (Kilpinen et al., 2013), and before and after adipogenic and osteogenic differentiation (Silva et al., 2020). However, discrimination between MSC and similar cell types such as fibroblasts based on their lipid profiles was not attempted so far.

On that basis, it appears intriguing to further investigate the potential of lipid analyses for MSC characterization. Due to their high amount in biological membranes, especially phospholipids are very promising lipid biomarkers (González-Domínguez, 2018). Previously, we have already established a mass spectrometry-based approach to monitor *de novo* synthesis of phospholipids in MSC by matrix-assisted laser desorption/ionization mass spectrometry (MALDI MS) (Prabutzki et al., 2020). However, since phospholipids exist in an enormous diversity, their detection is still challenging in crude mixtures. Therefore, we here used an untargeted liquid chromatography coupled to mass spectrometry (LC-MS) method, which allows the identification of a large number of lipids and is much more sensitive for detecting low abundant ones. The aim of the current study was to investigate whether lipid characterization by LC-MS is a useful tool to identify and discriminate MSC across culture conditions and species. Our hypothesis was that characteristic MSC lipid markers can be identified, which are sufficiently consistent between donors and across culture conditions to discriminate the MSC from other mesodermal cell types.

## Materials and Methods

### Cell Sources and Culture

Human MSC (hMSC; n = 5) were obtained from subcutaneous adipose tissue that was collected from waste material from waterjet-assisted liposuction in patients aged 31–45 years. Equine adipose-derived MSC (eMSC; n = 5) were obtained from subcutaneous adipose tissue that was harvested *en bloc* within the framework of an experimental animal study and from animals euthanized for unrelated reasons, with a donor age of 3–8 years. In addition, human scar tissue fibroblasts (hFibroblasts; n = 4) from donors with unknown age and human buffy coat PBMC (hPBMC; n = 5) from donors aged 20–71 years, obtained from discarded blood donations, were used. MSC and hFibroblasts were isolated by collagenase digestion and subsequent culture of plastic-adherent cells, as described previously (Hillmann et al., 2016). Aliquots of these cell types were subjected to conventional cell characterization including trilineage differentiation (Dominici et al., 2006) (Supplement information S1), before the remaining cells were cryopreserved.

For the current experiments, cells were thawed, seeded at a density of approximately 3,000 cells/cm^2^ and cultured in standard conditions until they reached 80% confluency. Standard conditions comprised culture in standard cell culture flasks in Dulbecco’s modified Eagle’s medium with 1 g Glucose/L (DMEM; Gibco™, ThermoFisher Scientific, Darmstadt, Germany), 10% fetal bovine serum (FBS; Gibco^™^, ThermoFisher Scientific; Lot: 2078409), 1% penicillin-streptomycin and 0.1% gentamycin, and incubation in a humidified atmosphere at 37°C and 5% CO_2_.

In order to estimate the influence of cell culture media on the MSC phenotype, hMSC from each donor were also cultured in a different medium previously optimized for hMSC culture. The latter consisted of *α* Minimum Essential Medium (αMEM; Gibco™, ThermoFisher Scientific) supplemented with 2.5% human platelet lysate (hPL; PL Bioscience GmbH, Aachen, Germany; Lot: 18-002.08), 1 U/ml heparin (Ratiopharm, Ulm, Germany) and antibiotics. Correspondingly, eMSC from each donor were also cultured with 10% equine buffy-coat-derived platelet lysate (ePL; Hagen et al., 2021; same batch used for all experiments) and 1 U/ml heparin instead of FBS, but in the same DMEM as specified above. Furthermore, in order to estimate the influence of an extracellular matrix environment on the MSC phenotype, hMSC were additionally cultured in collagen-coated flasks (Corning™ BioCoat™ Collagen I; purchased from VWR International), again using FBS-supplemented DMEM.

Following expansion in these different conditions, hMSC, eMSC and hFibroblasts at passage two or three were harvested by trypsinization, followed by washing steps in phosphate buffered saline. The cells from each donor were aliquoted individually for each culture condition and cell type, and stored at −80°C until lipid characterization.

Human PBMC were purified by density gradient centrifugation at 290 *g* for 45 min using Ficoll-Paque™ PREMIUM with a density of 1.077 g/ml (Cytiva Europe GmbH, Munich, Germany), washed and directly aliquoted for lipid characterization as described above.

### Lipid Characterization by Liquid Chromatography Coupled to Mass Spectrometry

All solvents and chemicals were purchased at highest commercially available purity and used without further purification. Methanol, 2-propanol and formic acid (all ULC/MS grade) were from Biosolve (Valkenswaard, Netherlands), whereas chloroform and ammonium formate were from Merck KGaA (Darmstadt, Germany).

#### Sample Preparation

Lipid extraction was achieved in glass vials according to Bligh and Dyer (1959) by adding 50 µL water followed by 100 µL chloroform/methanol (1/1, v/v) to the cell pellets (750,000 cells). After the samples were gently vortexed and centrifuged (10 min, 4°C, 10,000 x g), the lower chloroform phase was transferred to a new glass vial and evaporated to dryness. The evaporated samples were reconstituted in 50 µL propan-2-ol/methanol/water (5/1/4, v/v) containing 5 mM ammonium formate (NH_4_CO_2_H) and 0.2% formic acid (FA) for the following MS experiments.

#### LC - MS

LC-MS measurements were carried out on a Dionex Ultimate 3,000 high-performance liquid chromatography (HPLC) system (Thermo Fisher Scientific GmbH, Dreieich, Germany) coupled to an amaZon SL (Bruker Daltonics, Bremen, Germany) ion trap (IT) mass spectrometer equipped with an electrospray ionization (ESI) source.

Reversed phase (RP) LC was performed with a Kinetex C18 column (100 × 4.6 mm, 2.6 µm; Phenomenex, Aschaffenburg, Deutschland) using propan-2-ol/methanol/water (5/1/4, v/v) containing 5 mM NH_4_CO_2_H and 0.2% FA as eluent A and Propan-2-ol containing 5 mM NH_4_CO_2_H and 0.2% FA as eluent B. 20 µL of each extracted cell sample was separated by the following gradient: 0–0.2 min − 5% B, 5 min −8% B, 5.1 min −15% Β, 20 min −50% B, 21 min −90% B, 23 min −100% B, 26 min −100% B, 26.1 min −5% B, 28 min −5% B using a flow rate of 1 ml/min and a column temperature of 40°C.

The untargeted qualitative lipid analysis was done by tandem mass spectrometry (MS^2^) with alternating (positive and negative) ionization mode and the following source conditions: mass range mode: enhanced resolution; capillary voltage: 4,500 V; end plate offset: 500 V; nebulizer (nitrogen): 70 psi; dry gas: 12 L/min and dry temperature: 350°C. MS^2^ spectra, using a data dependent acquisition (DDA) of four precursor ions, were recorded by the “ion charge control” (ICC) and maximum acquisition time of 35,000 and 20 ms, respectively. The collision-induced dissociation (CID) parameters were set to: CID ramp: 50–250%, fragmentation time: 20 ms and fragmentation delay: 10 ms. Data acquisition and analysis were carried out by the tools “TrapControl” and “DataAnalysis”, respectively (Bruker Daltonics, Bremen, Germany).

Lipid identification was done manually according to their specific fragmentation pattern after CID experiments and was afterwards verified using both the LIPID MAPS^®^ database (The LIPID MAPS^®^ Lipidomics Gateway, https://www.lipidmaps.org/) and the ALEX software (Husen et al., 2013), which is available at www.msLipidomics.info. The exact lipid classification (ester-linked vs ether-linked phospholipids) was additionally verified by plotting the mass defect (difference between exact and nominal mass of one compound) against the double bond content of each lipid. The so-called Kendrick Mass Plots were done for the most abundant lipid classes in MSC, phosphatidylcholine (PC) and phosphatidylethanolamine (PE), according to Sleno (2018). Plotted mass defects against double bond content (see one example in Supplement information S2) should verify the correct identification of lipids against outliers which could be other lipid species as well.

#### Data Analysis

IBM SPSS Statistics 26 was used for comparisons between groups. Nonparametric tests were used for comparisons between paired samples from the different cell culture conditions as well as for group comparisons between the unpaired samples from the different cell types. If adequate, post hoc tests with Bonferroni correction were included. Differences were considered as significant at *p* ≤ 0.05.

### Results

#### Lipid Classes Identified in Mesodermal Cell Types

In general, using our untargeted lipidomics approach, we were able to identify 117 phospholipids and 26 sphingomyelin species in MSC. Lipids detected in hMSC, all three culture conditions taken together, comprised 36 phosphatidylcholine (PC) species, 30 phosphatidylethanolamine (PE) species, 12 phosphatidylglycerol (PG) species, 13 phosphatidylinositol (PI) species and 13 phosphatidylserine (PS) species, and at least 21 sphingomyelin (SM) species. Similarly, in eMSC, the two culture conditions taken together, 36 PC species, 29 PE species, 8 PG species, 10 PI species and 10 PS species, and at least 18 SM species were found. Less lipid diversity was observed in the other mesodermal cell types, hPBMC and hFibroblasts. In hPBMC, 24 PC species, 13 PE species, three PI species and 5 PS species, and at least 10 SM species were found. In hFibroblasts, lipids detected included 26 PC species, 19 PE species, eight PI species and 10 PS species, and at least 10 SM species. Remarkably, in contrast to the MSC, no PG was detectable in hPBMC and hFibroblasts. Other lipids such as di- and triacylglycerols or ceramides were below the detection threshold of the current LC-MSC method or absent in any of the samples analysed. Regarding phospholipid molecular species discrimination, it is of note that particularly in MSC, part of the phospholipids were detected at two different retention times. This was likely due to corresponding ether lipids also present in the samples, as ether lipids could not be finally discriminated with the untargeted analytic approach used, which is illustrated using PE 39:4 as an example in Supplement information S1.

**FIGURE 1:**
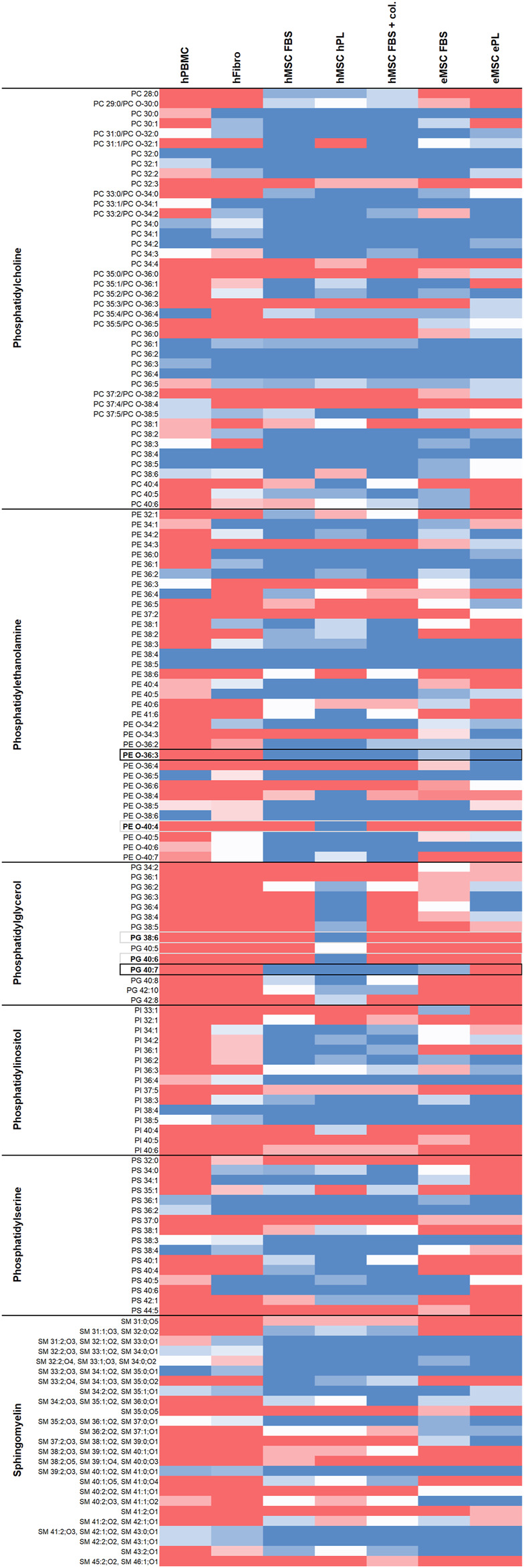
Overview of lipids detected in the different cell types and culture conditions. Dark blue color represents lipids detected by untargeted LC-MS in all respective donors, red color represents lipids found in none of the respective donors, while light colors indicate less consistency across donors (n = 4 for hFibroblasts, n = 5 for all other cell types). Phospholipid species detected at two retention times in at least one sample type, possibly due to corresponding ether lipids also present, are labeled with asterisks (*). Phospholipid species potentially suitable for inclusive MSC discrimination are highlighted with black borders, phospholipid species exclusively present in hMSC cultured with hPL are highlighted with grey borders.

Figure 1 gives an overview of the lipids detected in all cell types and culture conditions. Regarding phospholipid molecular species discrimination, it is of note that part of the PC species could not be finally discriminated as to whether they were ester lipids (1-acyl-2-acyl-phospholipids) or ether lipids (1-alkyl-2-acyl-phospholipids or 1-alkenyl-2-acyl-phospholipids). Therefore, both options are given for the respective PC (Figure 1). A detailed characterization of detected phospholipids including their theoretical *m/z* ratios and chemical formula can be found in Supplement information S3. For further analyses and comparisons between cell types or culture conditions, we focused on the phospholipids and their molecular species diversity.

#### Effect of MSC Culture Conditions on Phospholipid Diversity

**FIGURE 2:**
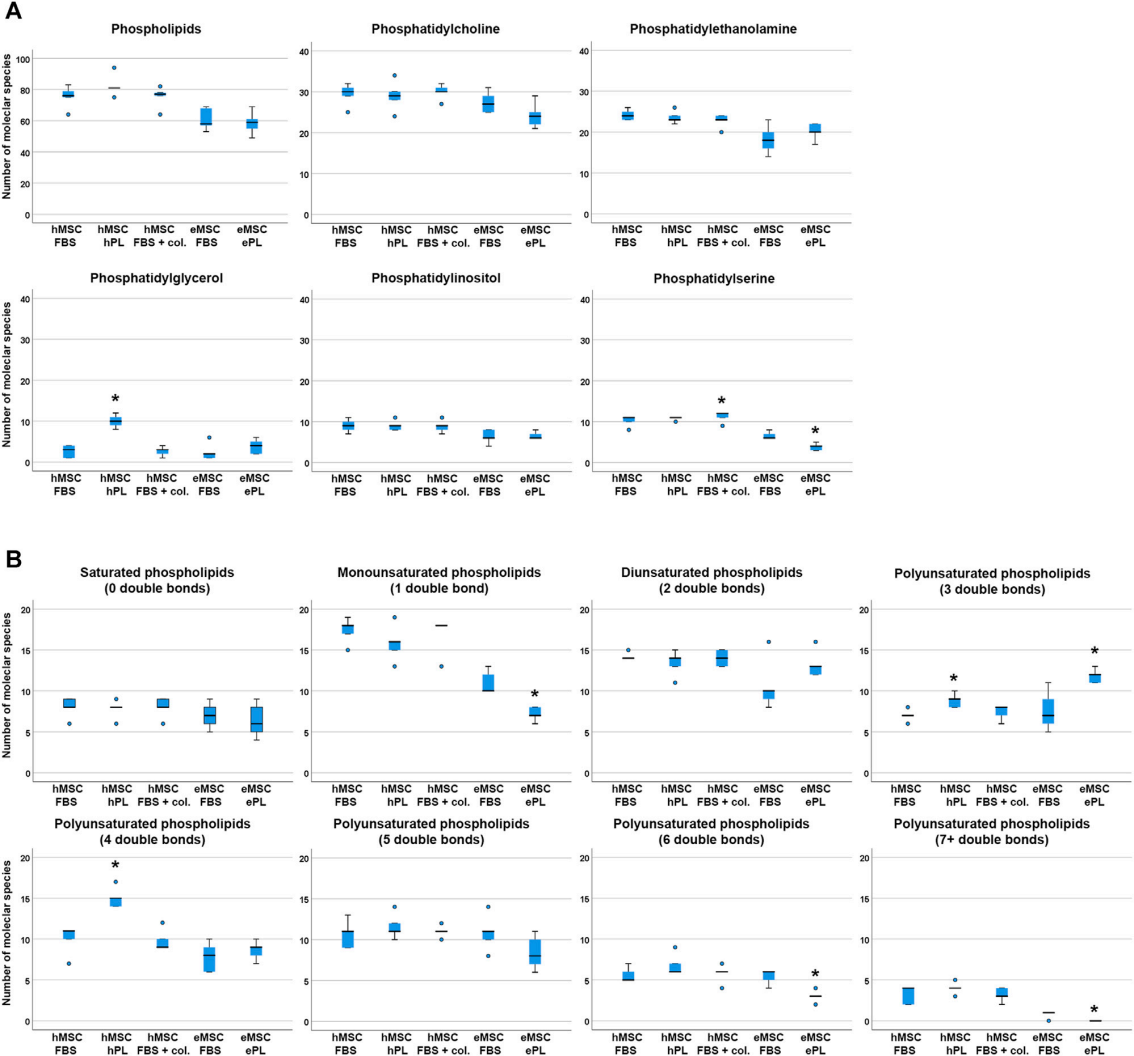
Phospholipid species diversity across MSC cell culture conditions. Panel A boxplots represent the numbers of either total phospholipid molecular species or the numbers of phospholipid species within the different phospholipid classes. Panel B boxplots represent the numbers of phospholipid molecular species within lipid groups by extent of saturation. Asterisks indicate significant differences (*p* ≤ 0.050) between the respective MSC group as compared to MSC from the same species cultured in standard conditions (i.e., with FBS). Data were obtained from n = 5 donors in all groups and donors from the same species were paired.

The total numbers of phospholipid species found in each sample did not differ significantly between culture conditions, neither in hMSC nor in eMSC (Figure 2). For more detailed analysis, we therefore compared the numbers of different phospholipid species within the different phospholipid classes (PC, PE, PG, PI and PS) and within lipids with a different extent of saturation, i.e. different numbers of double bonds, between hMSC or eMSC cultured in different conditions. Yet surprisingly, the culture conditions had relatively few effects on lipid diversity.

In the human cells, a higher number of different PG was found in hMSC cultured with hPL supplementation as compared to hMSC cultured with FBS (*p* = 0.027), and more different phospholipid species with either three or four double bonds were detected in hMSC cultured with hPL than in hMSC cultured with FBS (*p* = 0.011 and *p* = 0.027, respectively). Collagen coating led to a higher PS species diversity as compared to standard culture with FBS (*p* = 0.027), but no further differences were found due to this cell culture environment (Figure 2).

In the equine cells, a higher number of different PS was found in eMSC cultured with FBS supplementation as compared to eMSC cultured with ePL (*p* = 0.042). In addition, more different phospholipid species with either 1, six or seven double bonds were detected in eMSC cultured with FBS than in eMSC cultured with ePL (*p* < 0.050). Only within the phospholipids with three double bonds, more different lipid species were found in eMSC cultured with ePL (*p* = 0.042) (Figure 2).

#### Phospholipid Diversity in MSC and Other Mesodermal Cell Types

**FIGURE 3:**
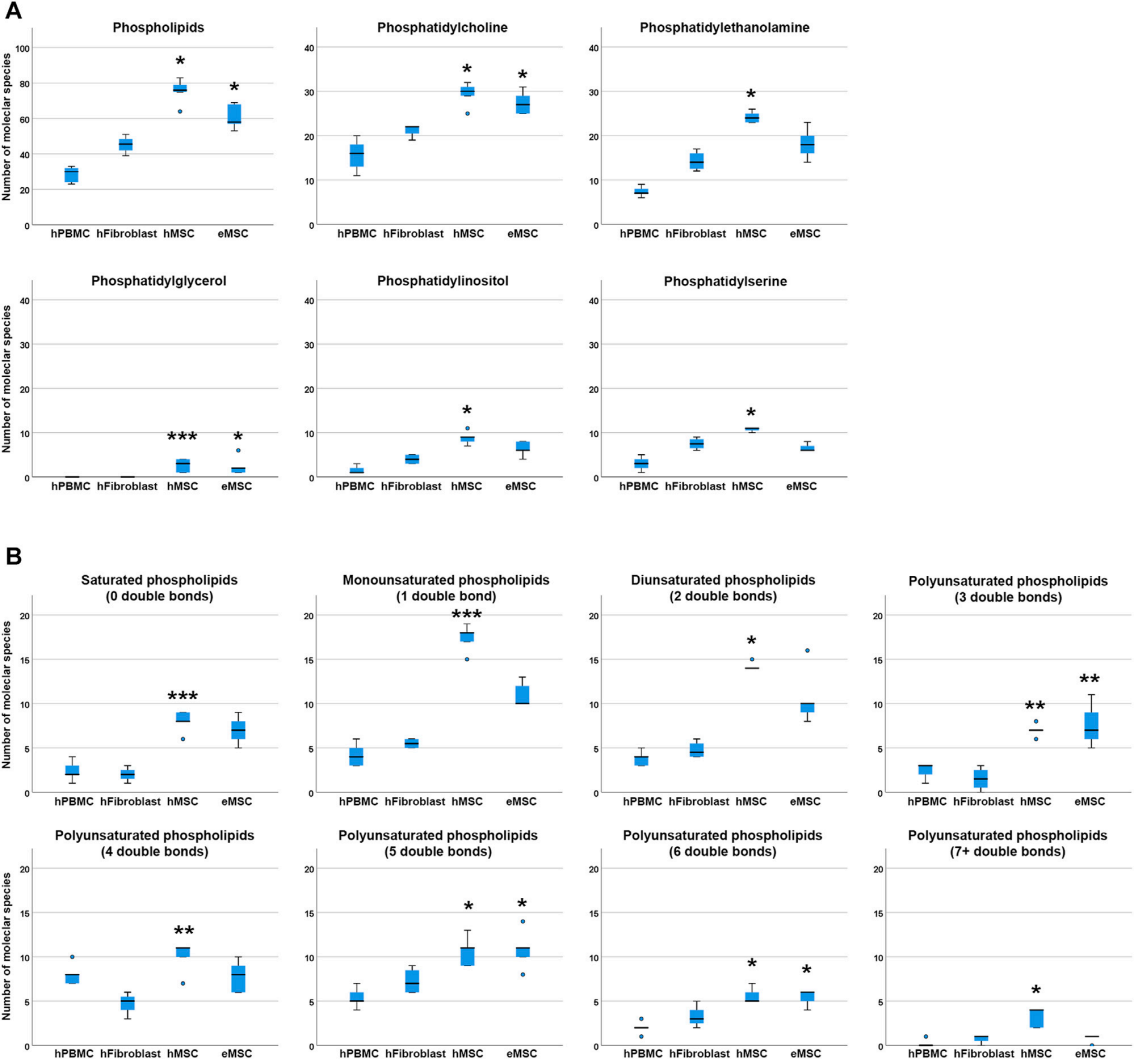
Phospholipid species diversity in different mesodermal cell types. Panel A boxplots represent the numbers of either total phospholipid molecular species or the numbers of phospholipid species within the different phospholipid classes. Panel B boxplots represent the numbers of phospholipid molecular species within lipid groups by extent of saturation. Asterisks indicate significant differences between MSC and hPBMC (*), hFibroblasts (**) or both (***) (*p* ≤ 0.050). Data were obtained from n = 4 donors for hFibroblasts and n = 5 donors for all other cell types.

For comparison of lipid diversity between the different mesodermal cell types analysed, only the MSC samples cultured in standard culture conditions with FBS supplementation, corresponding to the fibroblast culture conditions, were considered. Even when including only these standard culture MSC samples, significantly higher total phospholipid species diversity was observed in hMSC but also in eMSC compared to hPBMC (*p* = 0.001 and *p* = 0.050, respectively), while no differences were found between hMSC and eMSC (Figure 3).

Regarding molecular species diversity within phospholipid classes, more PC species were found in hMSC and eMSC than in hPBMC (*p* = 0.004 for hMSC, *p* = 0.033 for eMSC). Furthermore, more PE, PI and PS species were observed in hMSC as compared to hPBMC (*p* = 0.001 for PE, PI and PS). The differences in numbers of PC, PE, PI and PS species were not significant when comparing MSC to hFibroblasts. Yet importantly, while PG were found in each hMSC and eMSC sample, no PG were found in hPBMC and hFibroblasts at all (*p* = 0.020 for hMSC vs hPBMC, *p* = 0.035 for hMSC vs hFibroblasts, *p* = 0.036 for eMSC vs hPBMC) (Figure 3).

The number of double bonds in the phospholipids ranged from 0 to 10 in hMSC, and from 0 to seven in eMSC, hPBMC as well as hFibroblasts. Thus, phospholipids with more than seven double bonds only occurred in hMSC, yet not in all donors, and always within the PG class. Furthermore, differences in the numbers of different lipid species were evident among the phospholipids with either 0, 1, 2, 5, six and seven or more double bonds between hMSC and hPBMC (*p* < 0.05), and for the phospholipids with either five or six double bonds also for eMSC vs hPBMC (*p* = 0.015 and *p* = 0.017, respectively). The number of phospholipid species with no or one double bond additionally differed between hMSC and hFibroblasts (*p* = 0.020 and *p* = 0.031, respectively). Interestingly, in phospholipids with either three or four double bonds, differences between the numbers of lipid species were significant between hMSC and hFibroblasts (*p* = 0.029 and *p* = 0.004, respectively), and eMSC and hFibroblasts (*p* = 0.025 in lipids with three double bonds), but not compared to hPBMC (Figure 3).

Phospholipid chain lengths ranged from 28 to 42 carbon atoms in hMSC, from 29 to 40 in eMSC, and from 30 to 40 in hPBMC and hFibroblasts. Phospholipids with chain lengths of 28, 29, 41 or 42 carbon atoms only occurred in hMSC, but again not in all donors. Comparisons regarding phospholipid diversity within lipids with different chain lengths yielded similar results as within the phospholipid classes or lipids with different extent of saturation (data not shown).

#### Candidate Lipid Markers for Targeted Analysis in MSC Phenotyping

While overall higher lipid species diversity was associated with the MSC phenotype, we also aimed to identify phospholipids that might be suitable as specific markers to directly discriminate MSC from other mesodermal cells. Here we aimed to focus on lipid species that were consistently present in MSC and consistently lacking in hPBMC as well as in hFibroblasts, as highlighted in Figure 1.

Twenty-nine to 57 out of all 122 detected phospholipid molecular species were inconsistently present or lacking across the donors within one group (hPBMC: 24%; hFibroblasts: 34%; hMSC, FBS: 28%; hMSC, hPL: 30%; hMSC, FBS + collagen coating: 25%; eMSC, FBS: 47%; eMSC, ePL: 33%) and were therefore less suitable as candidate markers. Nevertheless, the remaining phospholipid species were either present or lacking across all donors within one group, with 16–61 out of the 122 phospholipid species being consistently detectable in all donors (hPBMC: 13%; hFibroblasts: 18%; hMSC, FBS: 47%; hMSC, hPL: 50%; hMSC, FBS + collagen coating: 49%; eMSC, FBS: 27%; eMSC, ePL: 30%).

39:4 or its corresponding ether lipid (Supplement information S2) PE O-40:4, PG 38:6 and PG 40:6 were consistently present in hMSC cultured with hPL, while lacking in hPBMC and hFibroblasts. However, these lipids were also lacking in hMSC cultured with FBS and in eMSC, suggesting that they are associated with a certain hMSC phenotype.

**FIGURE 4:**
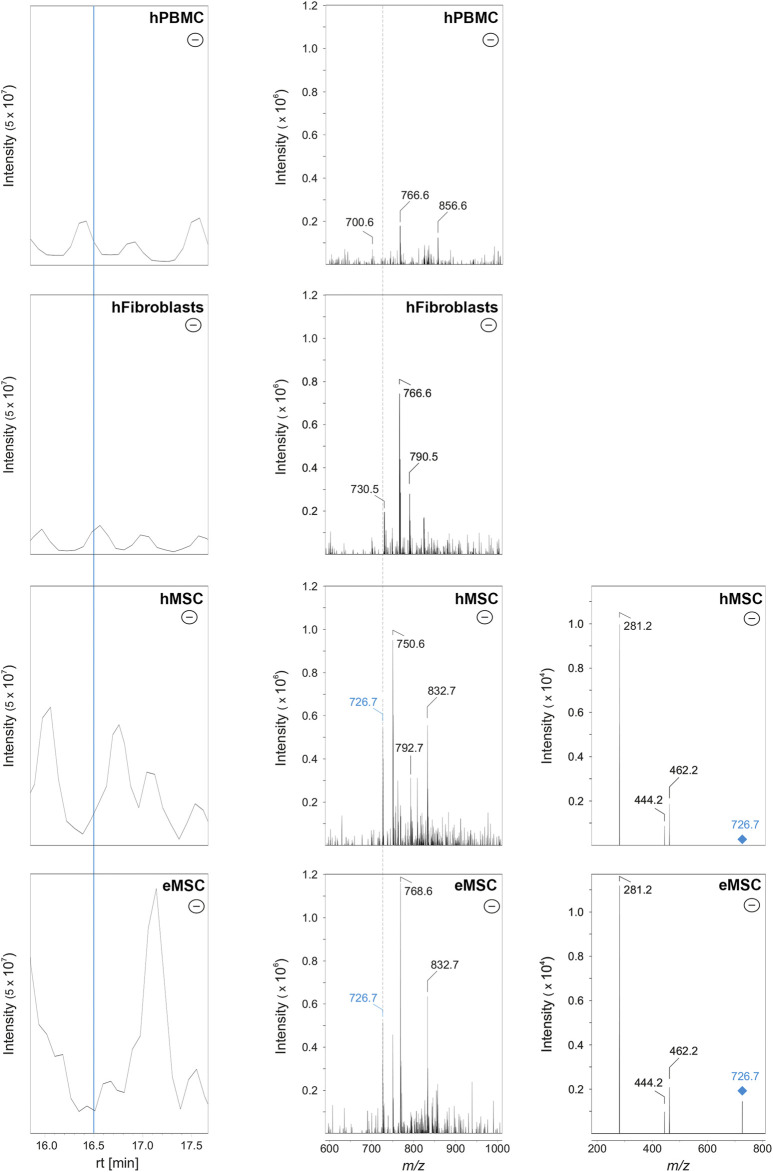
Identification of PE O-36:3 by liquid chromatography coupled to mass spectrometry in MSC. Left row: base peak chromatograms in the negative ionization mode of hPBMC, hFibroblasts, hMSC and eMSC in a retention time (rt) from 16.0 to 17.5 min. The MSC were cultured in the same standard medium with FBS as the hFibroblasts. The blue line indicates the rt at 16.5 min. Middle row: Corresponding mass spectra of hPBMC, hFibroblasts, hMSC and eMSC after negative ionization at rt 16.5 min showing a mass-to-charge (m/z) window from 600 to 1,000. The labels indicate those m/z values which were automatically picked for further tandem mass spectrometry (MS/MS) measurements (for further information see chapter: material and methods). Both the hMSC and eMSC samples lead to the signal with m/z 726.7 (labeled in blue). In hPBMC and hFibroblasts, signal at m/z 726.6 was completely absent (indicated by the dashed line). Right row: MS/MS spectra (negative ionization mode) of hMSC and eMSC of the precursor ion with m/z 726.6 (labeled in blue) which was fragmented by collision-induced dissociation (CID) leading to three fragment ions at m/z 462.2, 444.2 and 281.2. The signals at m/z 281.2 and 462.2 correspond to the free fatty acid 18:1 and lyso-PE P-18:1, respectively, leading to the identification of PE O-36:3. Some CID spectra of MSC samples showed also fragment ions matching free fatty acid 18:1 and lyso-PE P-18:1 as an additional fatty acid contribution (data not shown).

**FIGURE 5:**
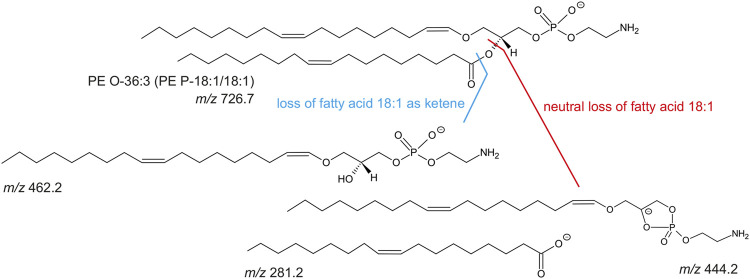
Molecular structure of PE O-36:3 (m/z 726.7 as [M-H]-) and known collision-induced dissociation fragment ions after negative ionization. The PE O-36:3 leads to the m/z 281.2 (free fatty acid 18:1), m/z 462.2 (lyso-PE P-18:1) after the loss of fatty acid 18:1 as ketene, or m/z 444.2 after the neutral loss of the fatty acid 18:1.

To discriminate MSC more globally, two phospholipid species and, alternatively, one phospholipid class appear most suitable. First, PE O-36:3, which was never present in hPBMC or hFibroblasts, but found in all hMSC samples and nine out of 10 eMSC samples, stands out as the most promising candidate marker that might discriminate MSC even across different species. Exemplary spectra and the underlying molecular structures are presented in Figures 4, 5. Second, PG 40:7 was present in all hMSC samples but not in any hPBMC or hFibroblast sample and could therefore be an additional candidate marker specific to hMSC. However, PG 40:7 was only found in four out of five eMSC samples cultured with FBS and in none of the eMSC samples cultured with ePL, thus its use as MSC marker across species is questionable. However, finally, the presence of any PG species might still be indicative of an MSC phenotype across species, as at least one PG was found in all MSC samples but not in hPBMC and hFibroblasts.

### Discussion

In the current study, we applied an untargeted lipidomics approach to identify lipid patterns that are useful as markers to discriminate MSC from other cell types, aiming to overcome the still existing challenge of proper MSC characterization due to the lack of known specific marker antigens. As the discrimination of MSC from cells from other lineages is generally less challenging, we focused on the differences between MSC and other mesodermal cell types, for which we chose hPBMC and hFibroblasts. Furthermore, we aimed at an inclusive approach for adipose-derived MSC from different species and culture conditions. As expected, the lipid phenotype of hPBMC differed strongly from all MSC groups analysed. However, intriguingly, we could also find differences between MSC and hFibroblasts, despite using exactly the same cell culture conditions prior to lipid analysis. At the same time, no differences were found between hMSC and eMSC when cultured in the same standard conditions, and relatively few differences between MSC cultured in different conditions. To the best of our knowledge, this is the first study that specifically focused on the MSC lipid phenotype, and we believe that it might be a starting point to discriminate MSC, despite their inevitable heterogeneity, from morphologically and immunophenotypically similar cells such as fibroblasts.

The main limitation of the untargeted lipidomics approach chosen is, in the first place, that the overall percentage of the respective lipids in the samples could not be quantified, since no reference standards were added during the lipid extraction procedure. To this day, only little is known about the lipid profile in MSC. Thus, we did not focus on the lipid quantification but on the identification of as many lipid species as possible. The second drawback is that not all isobaric lipids (1-acyl-2-acyl- vs 1-alkyl-2-acyl- (ether lipid) vs 1-alkenyl-2-acyl- (plasmalogen) phospholipids) could be unequivocally distinguished from each other, since tandem mass spectrometry experiments lead to the same fragment ions (Kim and Hoppel, 2020). This was true for PC and PE species with odd-chain carbon atoms. Since phospholipids with odd-chain fatty acids are uncommon (Sato et al., 2020), especially if they are unsaturated, and therefore not well-characterized, one might speculate that the odd-chain phospholipids could automatically be ether-linked lipids. To verify this theory, the fragmentation behavior of phospholipids in negative ionization mode was considered. As an example, PE 39:4 (ester PE) and PE O-40:4 (ether PE) both lead to the same precursor ion at *m/z* 780.56 in the negative ionization mode. Supplement Figure S2 (Panel A), illustrates the presence and fragmentation behavior of this precursor ion in hMSC cells cultivated with PL. Based on its MS/MS spectra alone, the identification of this PE is difficult. Therefore, the following was considered: 1) as indicated above, phospholipids with odd-chain fatty acids are uncommon, 2) ether lipids lead to only one free fatty acid after MS/MS, 3) cleavage of fatty acid as ketene leads to higher signal than the neutral loss of this fatty acid. In case of PE O-40:4 (Supplement Figure S2 (Panel C)), the ether linkage, which is typically in the sn*1* position at the glycerol backbone, leads to a lesser fragmentation efficiency (only 30%) than its corresponding ester lipid (Mitchell et al., 2007). Therefore, only the sn*2*-esterified fatty acid and its corresponding lyso-lipids could be found after MS/MS experiments (see Supplement Figure 2S (Panel A), and the structures in Supplement Figure 2S (Panel C)). Measuring only three fragments at *m/z* 333.2, 446.3 and 464.3 (as [M-H]^-^ in negative ionization mode) corresponding to the free fatty acid 22:3 (*m/z* 333.2), lyso-PE P-18:1 (*m/z* 464.3) and neutral loss of sn*2* fatty acid 22:3 from precursor ion (*m/z* 446.3), respectively, this PE species has to be PE O-40:4. Otherwise, in case of PE 39:4 (ester PE), corresponding fragment ions of the fatty acid 17:1 in sn*1*-position should have been detectable, too (for fragmentation behavior, see Supplement Figure S2 (Panel B)). Whereas the discrimination between ester lipids and ether lipids in PE could be unambiguously identified due to their fragmentation behavior in negative ionization mode, ester and ether discrimination of PC species could not be guaranteed using this method. Therefore, we decided to suggest both possible species (the ester and the isobaric ether) for questionable PC (Figure 1).

Since we found some of them in the analysed samples, for more detailed lipidomic cell characterization, it will have to be verified whether these lipids are ester-linked phospholipids, ether lipids or plasmalogens.

All analytical limitations will have to be addressed either by a targeted lipidomics approach including the respective internal standards for every phospholipid the candidate lipid markers, or at least with commercially available internal standard mixtures (like SPLASH® LIPIDOMICS®) including at least one non-native internal standard per lipid class, in the future. Internal standards (isotopically labeled, odd-numbered carbon atoms or absent in the sample) are known to show the same behavior in LC-MS approaches and have defined concentrations. Thus, they are useful to understand the chromatographic and mass spectrometric properties and allow the accurate quantification of unknown samples (Holčapek et al., 2018). Moreover, the performance of MS^3^ experiments for PC species could be helpful to identify ether lipids (Engel et al., 2017). Another approach to distinguish the lipid distribution in different cell types might be the analysis by matrix assisted laser/desorption ionization imaging mass spectrometry (MALDI-MSI), which allows the detection of lipids (and other biomolecules) in native samples without lipid extraction (Neumann et al., 2019a). Under certain conditions, this method allows even the analysis of single cells, as reported for astrocytes and neurons (Neumann et al., 2019b). Due to the combination of single cell MALDI MSI and immunocytochemistry, similarities and differences of the lipidome could be outlined without any information loss by harsh sample treatment steps. Yet so far, in the current analyses, we focused on the remarkable differences in phospholipid diversity, and on identifying candidate lipid markers for MSC.

Due to the well-known heterogeneity of MSC, we had expected to find considerable differences between hMSC and eMSC, as well as between MSC from the different culture conditions. This assumption was fostered by a previous study in which, based on the observed differences between the lipidomic profiles of tissues and cultured cells, the authors concluded to expect a strong impact of cell culture conditions on the cellular lipid profile (Symons et al., 2021). Yet favourably, we could find more differences between MSC and the other cell types than between MSC from the two species or culture conditions. Between human and equine cells, neither phospholipid species diversity differed significantly, nor was there any specific lipid consistently present in one species but not in the other. This is in accordance with previous studies, in which despite their different diets, the phospholipid composition in tissues or body fluids from different mammalian species was reported to be similar (Youngs and Corneatzer, 1963; Fuchs et al., 2009). Nevertheless, although not significant, lipid species diversity tended to be higher in hMSC than in eMSC. Furthermore, particularly in standard culture with FBS, the presence or lack of a certain lipid was less consistent across eMSC from different donors than in all other cell types, revealing higher heterogeneity in the eMSC and making them more difficult to discriminate. Comparing the different culture conditions, phospholipid species diversity was higher in hMSC cultured with hPL than with FBS, while the contrary was the case in eMSC. However, this was only true for very few phospholipid classes or groups, while the numbers of total phospholipid species found in each culture condition were similar. More remarkably, we found three phospholipid species, two of them PG, which were exclusively present in hMSC cultured with hPL. As these were not detected in the hPL culture medium (data not shown), they appear to be associated with a certain MSC phenotype. While hPL-supplemented medium is known to improve hMSC functionality (Bieback, 2013; Altaie et al., 2018; Czapla et al., 2019) and, as further discussed below, the presence of PG might be linked to functional properties, it could be speculated that this MSC phenotype may even be indicative of high potency. Yet overall, aside from the peculiaritis observed in hMSC cultured with hPL, all MSC phospholipid profiles were widely similar, raising hope for lipid markers applicable for MSC across species and culture conditions. Still, obviously, there are many other possible variations in cell culture conditions which may have an impact that remains to be investigated. Furthermore, differences between the lipid profiles of adipose- and muscle-derived MSC have already been described (Silva et al., 2020), and MSC from different tissue sources were not included in the current analyses. Future studies will have to further investigate and compare the phospholipid profiles in MSC from different tissue sources and animal species. This is also of importance with respect to confirming that the phenotype observed in the current study is truly related to the characteristics of MSC, rather than to their origin tissue.

MSC from both species strongly differed from hPBMC, due to their higher phospholipid diversity as well as based on several phospholipid species consistently lacking in hPBMC but present in MSC. This was not unexpected, but it is also of questionable usefulness, as PBMC and MSC are easily distinguished immunophenotypically, e.g. based on the pan-leukocyte marker CD45 which is absent in healthy MSC. Others previously proposed that a low percentage of PS might be indicative of MSC (Symons et al., 2021). However, in that previous work, red blood cells were also low in PS, and in the current study, PS species diversity was even lower in hPBMC and hFibroblasts. Therefore, a low PS percentage or low number of PS species might be useful to discriminate mesodermal cells from those of other lineages, but not from similar mesodermal cells, namely the fibroblasts.

In terms of discriminating MSC from fibroblasts, most importantly, we also identified lipid species consistently lacking in the hFibroblasts but present in MSC. Furthermore, differences in phospholipid species diversity became evident among the groups with either 0, 1, three or four double bonds, as well as within the PG headgroups that were lacking in hFibroblasts and hPBMC. Considering the extent of saturation, it was also remarkable that only in hMSC, highly unsaturated lipids with more than seven double bonds were found, again discriminating the hMSC from the hFibroblasts. This is in accordance with the finding that the percentage of polyunsaturated lipids with four or more double bonds was higher in bone marrow MSC than in most cell lines, such as CHO or MDCK cells (Symons et al., 2021). Interestingly, this study also showed that these polyunsaturated lipids were even more abundant in freshly isolated tissue cells, suggesting that loss of highly unsaturated lipids is a cell culture phenomenon due to a lack of these lipids in culture media (Symons et al., 2021). Yet we have previously shown that eMSC are capable of *de novo* synthesis of polyunsaturated phospholipids with at least four double bonds (Prabutzki et al., 2020). Potentially, the more naïve and not fully differentiated MSC have a higher synthetic capacity and are less susceptible to this loss than other cell types, but this remains speculative.

The lipid species which our findings highlighted as potential markers had either PE or PG headgroups. Previous findings highlighted lipid species with these headgroups, namely PE 32:1, PE 36:4, PE 40:1 and PG 38:4, as changing during MSC differentiation (Silva et al., 2020). Furthermore, certain PE species were shown to be increasing (PE 38:4) or decreasing (PE 36:2) with prolonged passaging, which is associated with a less potent phenotype, in human bone marrow MSC (Kilpinen et al., 2013). Another study showed that some lipids, including certain PI and PS, accumulate in dividing cells and their midbodies (Atilla-Gokcumen et al., 2014). The dividing cells had a highly specific lipid composition including many rare lipid species with widely unknown biological functions, suggesting that these specific lipids play key roles during cell cycle. Yet, these particular species were not among our most consistent and discriminative markers. Nevertheless, PE previously appeared to be overall associated with high proliferative capacity (Silva et al., 2020) and thus might be linked to stem cell-like properties.

PG, which we identified as the most discriminative phospholipid class in MSC, have so far received little attention as part of the MSC lipidomic profile. This is likely owed to their low abundance in mammalian cells (Poelma et al., 2005), which makes them less detectable depending on the analytic method. Besides their presence in procaryotes and plants, in mammals, PG are best known for their contribution to lung surfactant, although their implication in several essential other cellular processes has also been demonstrated (Furse, 2016). Important functions include its role in innate immunity, where PG can inhibit toll-like receptor activation and thereby decrease the inflammatory response (Choudhary et al., 2019; Voelker and Numata, 2019). At the same time, PG, together with PI, confine respiratory viral infection by preventing virus uptake by the host cells (Voelker and Numata, 2019). Other than in lung surfactant, PG appear to be mainly present in mitochondrial membranes, where they are used for the synthesis of cardiolipin. The latter is central to mitochondrial metabolism and alterations have been associated with disease such as diabetes or heart failure (Houtkooper and Vaz, 2008). Accordingly, PG supplementation was shown to decrease inflammation with downregulation of COX-2, and rescued mitochondrial activity in an *in vitro* inflammation model (Chen et al., 2018). Due to their anti-inflammatory and immunomodulatory properties, PG are also explored as therapeutic molecules which could be delivered by liposomes (Klein et al., 2021). In human bone marrow MSC, PG have previously been shown to be more abundant in preparations from whole cells than in the plasma membranes (Symons et al., 2021), which is in accordance with their role in mitochondrial membranes. Thus based on the roles of PG in mammalian cells, the presence of PG in MSC may not only be discriminative, but also be related to cellular functionality, such as mitochondrial density or immunomodulatory potential. Last not least, PG have been quantified by enzyme-based fluorometric assays (Morita and Terada, 2015), and such assays have become commercially available. They do not allow for the quantification of single PG species and can therefore not replace LC-MS/MS for detailed characterization of specific PG markers. However, given that PG content further crystallizes as distinctive and functional criterion for MSC characterization, this enzymatical method could easily be integrated in daily routine MSC characterization and complement the traditional approaches in the future.

## Data Availability

The original data presented in the study are included in the article/Supplementary Material, further inquiries can be directed to the corresponding authors.
