# Supplementary material for: Phospholipid Profiles for Phenotypic Characterization of Adipose-Derived Multipotent Mesenchymal Stromal Cells
Source: Front Cell Dev Biol. 2021 Dec 1;9:784405. doi: 10.3389/fcell.2021.784405 (PMC8672196; doi:10.3389/fcell.2021.784405)
Supplement: Supplementary file 1 [file DataSheet2.PDF]

Supplementary Material to Burk et al., Phospholipid-based MSC phenotyping

Supplement 2. Liquid chromatography coupled to mass spectrometry results and fragmentation patterns for PE 39:4 and its corresponding ether lipid, PE O-40:4.

S2 Supplementary Figure, panel A:
Upper row: Base peak chromatograms in the negative ionization mode of extracted human multipotent mesenchymal stromal cells (hMSC) from the same donor cultivated with fetal bovine serum (FBS, left), platelet lysate (PL, middle) or FBS + collagen coating (right) in a retention time (rt) from 19.0 to 21.0 min. The blue line indicates the rt at 19.8 min.
Middle row: Corresponding mass spectra after negative ionization at rt 19.8 min showing a mass-to-charge (*m/z*) window from 600 to 1000. The labels indicate those *m/z* values which were automatically picked for further tandem mass spectrometry (MS/MS) measurements (for further information see chapter: material and methods). Only the hMSC sample cultivated with PL leads to the signal with *m/z* 780.7 (labeled in blue).
Lower row: Spectrum on the left side shows the MS/MS spectrum (negative ionization mode) of the precursor ion with *m/z* 780.6 (labeled in blue), which was fragmentated by collision-induced dissociation (CID), leading to three dominant fragment ions at *m/z* 464.3, 446.3 and 333.2. The signals at *m/z* 333.2 and 464.3 correspond clearly to the free fatty acid (FFA) 22:3 and lyso-PE 17:1, respectively, leading to PE 39:4. Fragment ions marked with asterisks (*) could not be identified. The Kendrick Mass Plot on the right side could be used to verify the identified lipid species according to their unambiguous fatty acid assignment (for further details, see material and methods in the main text), showing all PE species with four double bonds. Whereas all identified PE species with even-numbered overall carbon atoms show a straight line, the two odd-numbered PE species are statistical outliers. This may indicate that PE 39:4 is actually no di-acyl esterified PE but rather an ether lipid (1-alkyl-2acyl PE) with the same molecular mass.

S2 Supplementary Figure, panel B:
Molecular structure of PE 39:4 (*m/z* 780.7) and known CID fragment ions after negative ionization. Theoretically, PE 39:4 leads to six different fragments: *m/z* 508.2 (FFA 17:1), *m/z* 512.3 (neutral loss of FFA 17:1), m/z 530.3 (lyso-PE 22:3), *m/z* 464.3 (lyso-PE 17:1), *m/z* 333.2 (FFA 22:3) or *m/z* 446.3 after the neutral loss of the sn2 fatty acid 22:3. S2 Supplementary Figure, panel C: Molecular structure of corresponding PE O-40:4 (*m/z* 780.7) which leads to the same fragment ions after negative ionization: *m/z* 464.3 (lyso-PE O-18:1), *m/z* 333.2 (FFA 22:3) and *m/z* 446.3 after the neutral loss of the sn2 fatty acid 22:3.

S2 Supplementary Figure, panel C:
Molecular structure of corresponding PE O-40:4 (*m/z* 780.7) which leads to the same fragment ions after negative ionization: *m/z* 464.3 (lyso-PE O-18:1), *m/z* 333.2 (FFA 22:3) and *m/z* 446.3 after the neutral loss of the sn2 fatty acid 22:3.

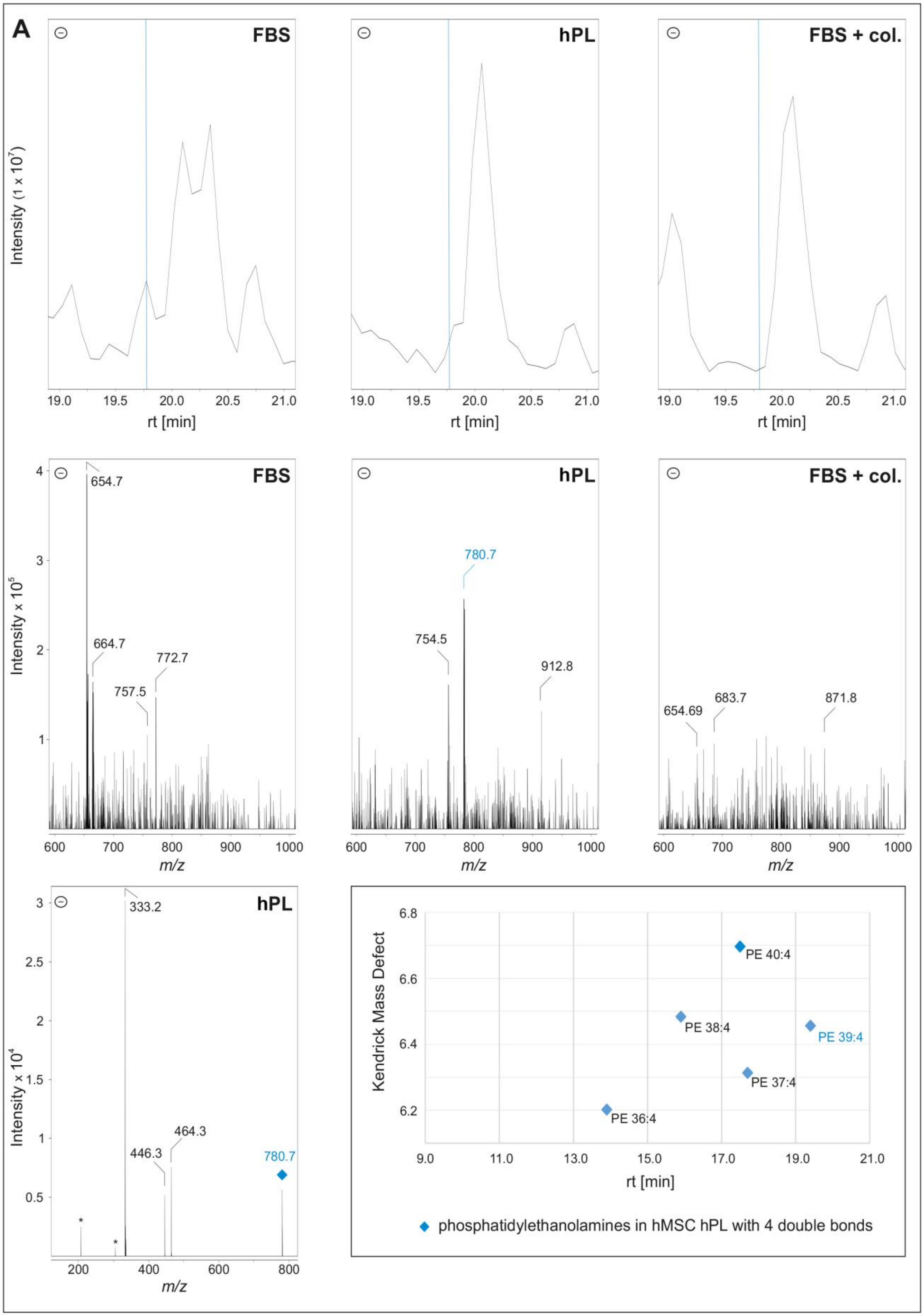
A
FBS
hPL
FBS + col.
Intensity (1 x 10^7)
rt [min]
19.0
19.5
20.0
20.5
21.0
Intensity x 10^5
654.7
664.7
772.7
757.5
780.7
754.5
912.8
654.69
683.7
871.8
m/z
Intensity x 10^4
333.2
464.3
446.3
780.7
Kendrick Mass Defect
PE 40:4
PE 38:4
PE 39:4
PE 37:4
PE 36:4
rt [min]
phosphatidylethanolamines in hMSC hPL with 4 double bonds

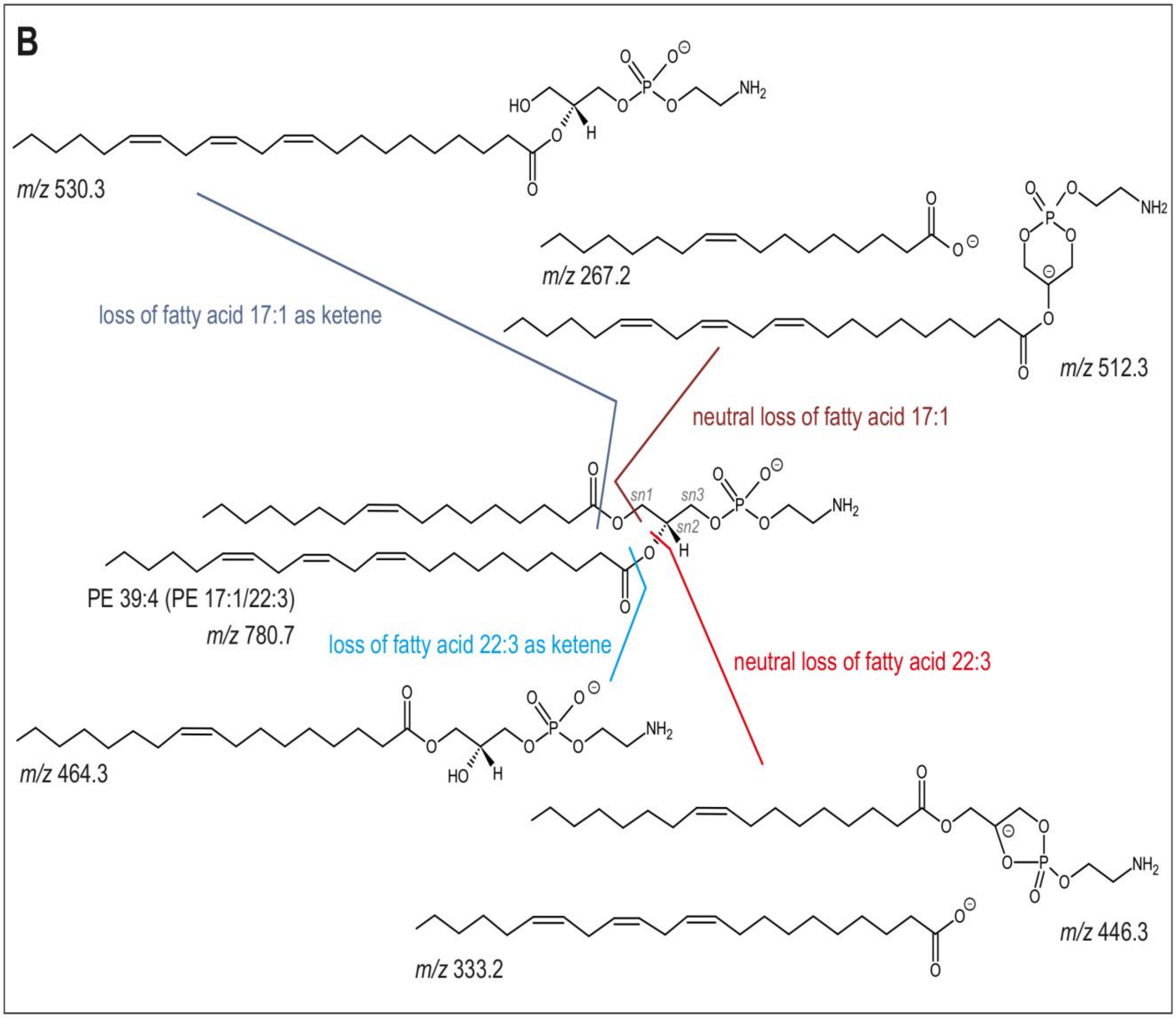
B
m/z 530.3
m/z 267.2
loss of fatty acid 17:1 as ketene
m/z 512.3
neutral loss of fatty acid 17:1
sn1
sn3
sn2
PE 39:4 (PE 17:1/22:3)
m/z 780.7
loss of fatty acid 22:3 as ketene
neutral loss of fatty acid 22:3
m/z 464.3
m/z 446.3
m/z 333.2

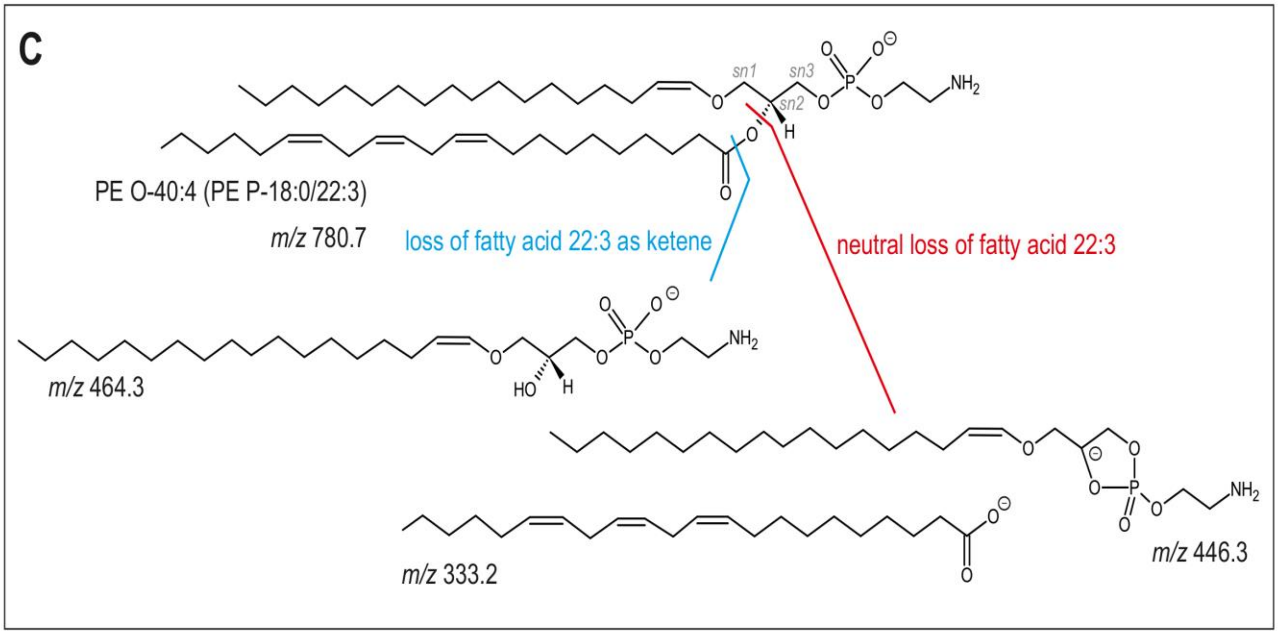
C
sn1
sn3
sn2
PE O-40:4 (PE P-18:0/22:3)
m/z 780.7
loss of fatty acid 22:3 as ketene
neutral loss of fatty acid 22:3
m/z 464.3
m/z 446.3
m/z 333.2
