# Supplementary material for: Phospholipid Profiles for Phenotypic Characterization of Adipose-Derived Multipotent Mesenchymal Stromal Cells
Source: Front Cell Dev Biol. 2021 Dec 1;9:784405. doi: 10.3389/fcell.2021.784405 (PMC8672196; doi:10.3389/fcell.2021.784405)
Supplement: Supplementary file 2 [file DataSheet3.PDF]

Supplementary Material to Burk et al., Phospholipid-based MSC phenotyping

Supplement 3: Overview and assignment of phospholipids.

The S3 Supplementary Table displays the assignment of phospholipids in positive and negative ionization mode using reversed-phase liquid chromatography coupled to electrospray ionization mass spectrometry and tandem mass spectrometry (MS/MS) experiments.

Lipid Species	Accurate m/z value	Adduct	Empirical Formula
**1-Acyl-2-acyl-phosphatidylcholine (PC)**		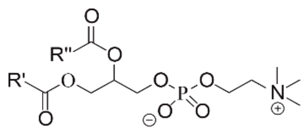	
**PC 28:0**	678.5068	$[M+H]^+$	$C_{36}H_{73}NO_8P$
	722.4978	$[M+HCOO]^-$	$C_{37}H_{73}NO_{10}P$
**PC 29:0**	692.5225	$[M+H]^+$	$C_{37}H_{75}NO_8P$
	736.5134	$[M+HCOO]^-$	$C_{38}H_{75}NO_{10}P$
**PC 30:0**	706.5381	$[M+H]^+$	$C_{38}H_{77}NO_8P$
	750.5291	$[M+HCOO]^-$	$C_{39}H_{77}NO_{10}P$
**PC 30:1**	704.5225	$[M+H]^+$	$C_{38}H_{75}NO_8P$
	748.5134	$[M+HCOO]^-$	$C_{39}H_{75}NO_{10}P$
**PC 31:0**	720.5538	$[M+H]^+$	$C_{39}H_{79}NO_8P$
	764.5447	$[M+HCOO]^-$	$C_{40}H_{79}NO_{10}P$
**PC 31:1**	718.5381	$[M+H]^+$	$C_{39}H_{77}NO_8P$
	762.5291	$[M+HCOO]^-$	$C_{40}H_{77}NO_{10}P$
**PC 32:0**	734.5694	$[M+H]^+$	$C_{40}H_{81}NO_8P$
	778.5604	$[M+HCOO]^-$	$C_{41}H_{81}NO_{10}P$
**PC 32:1**	732.5538	$[M+H]^+$	$C_{40}H_{79}NO_8P$
	776.5447	$[M+HCOO]^-$	$C_{41}H_{79}NO_{10}P$
**PC 32:2**	730.5381	$[M+H]^+$	$C_{40}H_{77}NO_8P$
	774.5291	$[M+HCOO]^-$	$C_{41}H_{77}NO_{10}P$
**PC 32:3**	728.5225	$[M+H]^+$	$C_{40}H_{75}NO_8P$
	772.5134	$[M+HCOO]^-$	$C_{41}H_{75}NO_{10}P$
**PC 33:1**	746.5694	$[M+H]^+$	$C_{41}H_{81}NO_8P$
	790.5603	$[M+HCOO]^-$	$C_{42}H_{81}NO_{10}P$
**PC 33:2**	744.5537	$[M+H]^+$	$C_{41}H_{79}NO_8P$
	788.5447	$[M+HCOO]^-$	$C_{42}H_{79}NO_{10}P$
**PC 34:0**	762.6007	$[M+H]^+$	$C_{42}H_{85}NO_8P$
	806.5916	$[M+HCOO]^-$	$C_{43}H_{85}NO_{10}P$
**PC 34:1**	760.5850	$[M+H]^+$	$C_{42}H_{83}NO_8P$
	804.5760	$[M+HCOO]^-$	$C_{43}H_{83}NO_{10}P$

Lipid Species	Accurate *m/z* value	Adduct	Empirical Formula
**PC 34:2**	758.5694<br>802.5603	$[M+H]^+$<br>$[M+HCOO]^-$	$C_{42}H_{81}NO_8P$<br>$C_{43}H_{81}NO_{10}P$
**PC 34:3**	756.5537<br>800.5447	$[M+H]^+$<br>$[M+HCOO]^-$	$C_{42}H_{79}NO_8P$<br>$C_{43}H_{79}NO_{10}P$
**PC 35:0**	776.6163<br>820.6073	$[M+H]^+$<br>$[M+HCOO]^-$	$C_{43}H_{87}NO_8P$<br>$C_{44}H_{87}NO_{10}P$
**PC 35:2**	772.5850<br>816.5760	$[M+H]^+$<br>$[M+HCOO]^-$	$C_{43}H_{83}NO_8P$<br>$C_{44}H_{83}NO_{10}P$
**PC 35:3**	770.5694<br>814.5603	$[M+H]^+$<br>$[M+HCOO]^-$	$C_{43}H_{81}NO_8P$<br>$C_{44}H_{81}NO_{10}P$
**PC 35:4**	768.5537<br>812.5447	$[M+H]^+$<br>$[M+HCOO]^-$	$C_{43}H_{79}NO_8P$<br>$C_{44}H_{79}NO_{10}P$
**PC 36:0**	790.6320<br>834.6229	$[M+H]^+$<br>$[M+HCOO]^-$	$C_{44}H_{89}NO_8P$<br>$C_{45}H_{89}NO_{10}P$
**PC 36:1**	788.6163<br>832.6073	$[M+H]^+$<br>$[M+HCOO]^-$	$C_{44}H_{87}NO_8P$<br>$C_{45}H_{87}NO_{10}P$
**PC 36:2**	786.6007<br>830.5916	$[M+H]^+$<br>$[M+HCOO]^-$	$C_{44}H_{85}NO_8P$<br>$C_{45}H_{85}NO_{10}P$
**PC 36:3**	784.5850<br>828.5760	$[M+H]^+$<br>$[M+HCOO]^-$	$C_{44}H_{83}NO_8P$<br>$C_{45}H_{83}NO_{10}P$
**PC 36:4**	782.5694<br>826.5603	$[M+H]^+$<br>$[M+HCOO]^-$	$C_{44}H_{81}NO_8P$<br>$C_{45}H_{81}NO_{10}P$
**PC 36:5**	780.5537<br>824.5447	$[M+H]^+$<br>$[M+HCOO]^-$	$C_{44}H_{79}NO_8P$<br>$C_{45}H_{79}NO_{10}P$
**PC 37:2**	800.6163<br>844.6073	$[M+H]^+$<br>$[M+HCOO]^-$	$C_{45}H_{87}NO_8P$<br>$C_{46}H_{87}NO_{10}P$
**PC 37:4**	796.5850<br>840.5760	$[M+H]^+$<br>$[M+HCOO]^-$	$C_{45}H_{83}NO_8P$<br>$C_{46}H_{83}NO_{10}P$
**PC 37:5**	794.5694<br>838.5603	$[M+H]^+$<br>$[M+HCOO]^-$	$C_{45}H_{81}NO_8P$<br>$C_{46}H_{81}NO_{10}P$
**PC 38:1**	816.6476<br>860.6386	$[M+H]^+$<br>$[M+HCOO]^-$	$C_{46}H_{91}NO_8P$<br>$C_{47}H_{91}NO_{10}P$
**PC 38:2**	814.6320<br>858.6229	$[M+H]^+$<br>$[M+HCOO]^-$	$C_{46}H_{89}NO_8P$<br>$C_{47}H_{89}NO_{10}P$
**PC 38:3**	812.6163<br>856.6073	$[M+H]^+$<br>$[M+HCOO]^-$	$C_{46}H_{87}NO_8P$<br>$C_{47}H_{87}NO_{10}P$
**PC 38:4**	810.6007<br>854.5916	$[M+H]^+$<br>$[M+HCOO]^-$	$C_{46}H_{85}NO_8P$<br>$C_{47}H_{85}NO_{10}P$
**PC 38:5**	808.5850<br>852.5760	$[M+H]^+$<br>$[M+HCOO]^-$	$C_{46}H_{83}NO_8P$<br>$C_{47}H_{83}NO_{10}P$
**PC 38:6**	806.5694<br>850.5603	$[M+H]^+$<br>$[M+HCOO]^-$	$C_{46}H_{81}NO_8P$<br>$C_{47}H_{81}NO_{10}P$

Lipid Species	Accurate *m/z* value	Adduct	Empirical Formula
**PC 40:4**	838.6320<br>882.6229	$[M+H]^+$<br>$[M+HCOO]^-$	$C_{48}H_{89}NO_8P$<br>$C_{49}H_{89}NO_{10}P$
**PC 40:5**	836.6163<br>880.6073	$[M+H]^+$<br>$[M+HCOO]^-$	$C_{48}H_{87}NO_8P$<br>$C_{49}H_{87}NO_{10}P$
**PC 40:6**	834.6007<br>878.5916	$[M+H]^+$<br>$[M+HCOO]^-$	$C_{48}H_{85}NO_8P$<br>$C_{49}H_{85}NO_{10}P$
**1-Alkyl-2-acyl-phosphatidylcholine (PC-O)**		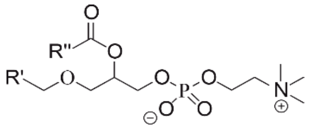	
**PC O-30:0**	692.5589<br>736.5498	$[M+H]^+$<br>$[M+HCOO]^-$	$C_{38}H_{79}NO_7P$<br>$C_{39}H_{79}NO_9P$
**PC O-32:0**	720.5902<br>764.5811	$[M+H]^+$<br>$[M+HCOO]^-$	$C_{40}H_{83}NO_7P$<br>$C_{41}H_{83}NO_9P$
**PC O-34:0**	748.6214<br>792.6123	$[M+H]^+$<br>$[M+HCOO]^-$	$C_{42}H_{87}NO_7P$<br>$C_{43}H_{87}NO_9P$
**PC O-34:1**	746.6058<br>790.5967	$[M+H]^+$<br>$[M+HCOO]^-$	$C_{42}H_{85}NO_7P$<br>$C_{43}H_{85}NO_9P$
**PC O-34:2**	744.5901<br>788.5810	$[M+H]^+$<br>$[M+HCOO]^-$	$C_{42}H_{83}NO_7P$<br>$C_{43}H_{83}NO_9P$
**PC O-36:2**	772.6214<br>816.6123	$[M+H]^+$<br>$[M+HCOO]^-$	$C_{44}H_{87}NO_7P$<br>$C_{45}H_{87}NO_9P$
**PC O-36:3**	770.6058<br>814.5967	$[M+H]^+$<br>$[M+HCOO]^-$	$C_{44}H_{85}NO_7P$<br>$C_{45}H_{85}NO_9P$
**PC O-36:4**	768.5901<br>812.5810	$[M+H]^+$<br>$[M+HCOO]^-$	$C_{44}H_{83}NO_7P$<br>$C_{45}H_{83}NO_9P$
**PC O-38:2**	800.6527<br>844.6436	$[M+H]^+$<br>$[M+HCOO]^-$	$C_{46}H_{91}NO_7P$<br>$C_{47}H_{91}NO_9P$
**PC O-38:4**	796.6214<br>840.6123	$[M+H]^+$<br>$[M+HCOO]^-$	$C_{46}H_{87}NO_7P$<br>$C_{47}H_{87}NO_9P$
**PC O-38:5**	794.6058<br>838.5967	$[M+H]^+$<br>$[M+HCOO]^-$	$C_{46}H_{85}NO_7P$<br>$C_{47}H_{85}NO_9P$
**1-Acyl-2-acyl-phosphatidylethanolamine (PE)**		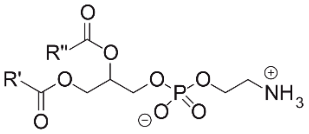	
**PE 32:1**	688.4922	$[M-H]^-$	$C_{37}H_{71}NO_8P$
**PE 34:1**	716.5235	$[M-H]^-$	$C_{39}H_{75}NO_8P$
**PE 34:2**	714.5079	$[M-H]^-$	$C_{39}H_{73}NO_8P$
**PE 34:3**	712.4922	$[M-H]^-$	$C_{39}H_{71}NO_8P$
**PE 36:0**	746.5705	$[M-H]^-$	$C_{41}H_{81}NO_8P$
**PE 36:1**	744.5548	$[M-H]^-$	$C_{41}H_{79}NO_8P$

Lipid Species	Accurate *m/z* value	Adduct	Empirical Formula
**PE 36:2**	742.5392	$[M-H]^-$	$C_{41}H_{77}NO_8P$
**PE 36:3**	740.5235	$[M-H]^-$	$C_{41}H_{75}NO_8P$
**PE 36:4**	738.5079	$[M-H]^-$	$C_{41}H_{73}NO_8P$
**PE 36:5**	736.4922	$[M-H]^-$	$C_{41}H_{71}NO_8P$
**PE 38:1**	772.5861	$[M-H]^-$	$C_{43}H_{83}NO_8P$
**PE 38:2**	770.5705	$[M-H]^-$	$C_{43}H_{81}NO_8P$
**PE 38:3**	768.5548	$[M-H]^-$	$C_{43}H_{79}NO_8P$
**PE 38:4**	766.5392	$[M-H]^-$	$C_{43}H_{77}NO_8P$
**PE 38:5**	764.5235	$[M-H]^-$	$C_{43}H_{75}NO_8P$
**PE 38:6**	762.5079	$[M-H]^-$	$C_{43}H_{73}NO_8P$
**PE 40:4**	794.5705	$[M-H]^-$	$C_{45}H_{81}NO_8P$
**PE 40:5**	792.5548	$[M-H]^-$	$C_{45}H_{79}NO_8P$
**PE 40:6**	790.5392	$[M-H]^-$	$C_{45}H_{77}NO_8P$
**1-Alkyl-2-acyl-phosphatidylethanolamine (PE-O)**			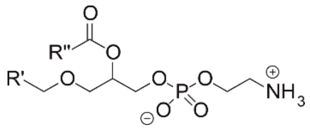
**PE O-34:2**	700.5286	$[M-H]^-$	$C_{39}H_{75}NO_7P$
**PE O-34:3**	698.5130	$[M-H]^-$	$C_{39}H_{73}NO_7P$
**PE O-36:2**	728.5599	$[M-H]^-$	$C_{41}H_{79}NO_7P$
**PE O-36:3**	726.5443	$[M-H]^-$	$C_{41}H_{77}NO_7P$
**PE O-36:4**	724.5286	$[M-H]^-$	$C_{41}H_{75}NO_7P$
**PE O-36:5**	722.5130	$[M-H]^-$	$C_{41}H_{73}NO_7P$
**PE O-36:6**	720.4973	$[M-H]^-$	$C_{41}H_{71}NO_7P$
**PE O-38:2**	756.5912	$[M-H]^-$	$C_{43}H_{83}NO_7P$
**PE O-38:4**	752.5599	$[M-H]^-$	$C_{43}H_{79}NO_7P$
**PE O-38:5**	750.5443	$[M-H]^-$	$C_{43}H_{77}NO_7P$
**PE O-38:6**	748.5286	$[M-H]^-$	$C_{43}H_{75}NO_7P$
**PE O-40:4**	780.5912	$[M-H]^-$	$C_{45}H_{83}NO_7P$
**PE O-40:5**	778.5756	$[M-H]^-$	$C_{45}H_{81}NO_7P$
**PE O-40:6**	776.5599	$[M-H]^-$	$C_{45}H_{79}NO_7P$
**PE O-40:7**	774.5443	$[M-H]^-$	$C_{45}H_{77}NO_7P$
**1-Acyl-2-acyl-phosphatidylglycerol (PG)**			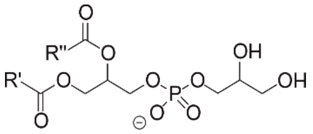
**PG 34:2**	745.5025	$[M-H]^-$	$C_{40}H_{74}O_{10}P$
**PG 36:1**	775.5494	$[M-H]^-$	$C_{42}H_{80}O_{10}P$
**PG 36:2**	773.5338	$[M-H]^-$	$C_{42}H_{78}O_{10}P$
**PG 36:3**	771.5181	$[M-H]^-$	$C_{42}H_{76}O_{10}P$

Lipid Species	Accurate *m/z* value	Adduct	Empirical Formula
**PG 36:4**	769.5025	$[M-H]^-$	$C_{42}H_{74}O_{10}P$
**PG 38:4**	797.5338	$[M-H]^-$	$C_{44}H_{78}O_{10}P$
**PG 38:5**	795.5181	$[M-H]^-$	$C_{44}H_{76}O_{10}P$
**PG 38:6**	793.5025	$[M-H]^-$	$C_{44}H_{74}O_{10}P$
**PG 40:5**	823.5494	$[M-H]^-$	$C_{46}H_{80}O_{10}P$
**PG 40:6**	821.5338	$[M-H]^-$	$C_{46}H_{78}O_{10}P$
**PG 40:7**	819.5181	$[M-H]^-$	$C_{46}H_{76}O_{10}P$
**PG 40:8**	817.5025	$[M-H]^-$	$C_{46}H_{74}O_{10}P$
**PG 42:8**	845.5338	$[M-H]^-$	$C_{48}H_{78}O_{10}P$
**PG 42:10**	841.5025	$[M-H]^-$	$C_{48}H_{74}O_{10}P$
**1-Acyl-2-acyl-phosphatidylinositol (PI)**		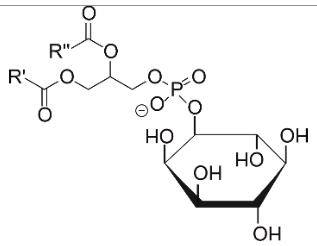	
**PI 32:1**	807.5029	$[M-H]^-$	$C_{41}H_{76}O_{13}P$
**PI 34:1**	835.5342	$[M-H]^-$	$C_{43}H_{80}O_{13}P$
**PI 34:2**	833.5185	$[M-H]^-$	$C_{43}H_{78}O_{13}P$
**PI 36:1**	863.5655	$[M-H]^-$	$C_{45}H_{84}O_{13}P$
**PI 36:2**	861.5498	$[M-H]^-$	$C_{45}H_{82}O_{13}P$
**PI 36:3**	859.5342	$[M-H]^-$	$C_{45}H_{80}O_{13}P$
**PI 36:4**	857.5185	$[M-H]^-$	$C_{45}H_{78}O_{13}P$
**PI 38:3**	887.5655	$[M-H]^-$	$C_{47}H_{84}O_{13}P$
**PI 38:4**	885.5498	$[M-H]^-$	$C_{47}H_{82}O_{13}P$
**PI 38:5**	883.5342	$[M-H]^-$	$C_{47}H_{80}O_{13}P$
**PI 40:5**	911.5655	$[M-H]^-$	$C_{49}H_{84}O_{13}P$
**PI 40:6**	909.5498	$[M-H]^-$	$C_{49}H_{82}O_{13}P$
**1-Acyl-2-acyl-phosphatidylserine (PS)**		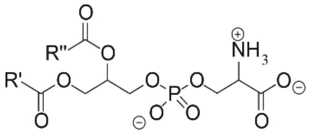	
**PS 32:0**	734.4977	$[M-H]^-$	$C_{38}H_{73}NO_{10}P$
**PS 34:0**	762.5290	$[M-H]^-$	$C_{40}H_{77}NO_{10}P$
**PS 34:1**	760.5134	$[M-H]^-$	$C_{40}H_{75}NO_{10}P$
**PS 36:1**	788.5447	$[M-H]^-$	$C_{42}H_{79}NO_{10}P$
**PS 36:2**	786.5290	$[M-H]^-$	$C_{42}H_{77}NO_{10}P$
**PS 38:1**	816.5760	$[M-H]^-$	$C_{44}H_{83}NO_{10}P$
**PS 38:3**	812.5447	$[M-H]^-$	$C_{44}H_{79}NO_{10}P$
**PS 38:4**	810.5290	$[M-H]^-$	$C_{44}H_{77}NO_{10}P$

Lipid Species	Accurate *m/z* value	Adduct	Empirical Formula
**PS 40:1**	844.6073	$[M-H]^-$	$C_{46}H_{87}NO_{10}P$
**PS 40:4**	838.5603	$[M-H]^-$	$C_{46}H_{81}NO_{10}P$
**PS 40:5**	836.5447	$[M-H]^-$	$C_{46}H_{79}NO_{10}P$
**PS 40:6**	834.5290	$[M-H]^-$	$C_{46}H_{77}NO_{10}P$
**PS 42:1**	872.6386	$[M-H]^-$	$C_{48}H_{91}NO_{10}P$
**PS 44:5**	892.6073	$[M-H]^-$	$C_{50}H_{88}NO_{10}P$
