# Supplementary material for: Phospholipid Profiles for Phenotypic Characterization of Adipose-Derived Multipotent Mesenchymal Stromal Cells
Source: Front Cell Dev Biol. 2021 Dec 1;9:784405. doi: 10.3389/fcell.2021.784405 (PMC8672196; doi:10.3389/fcell.2021.784405)

Supplementary Material to Burk et al., Phospholipid-based MSC phenotyping

Supplement 1. Characterization of MSC and fibroblasts.

<u>MSC characterization:</u>

Representative samples of human and equine MSC were characterized by immunophenotyping and trilineage differentiation before being included in the lipid phenotyping study. For more detailed characterization of equine and human MSC, as well as for detailed methods descriptions, please refer to Burk et al., 2013 (doi: 10.1016/j.tvjl.2012.06.004), Paebst et al., 2014 (doi: 10.1002/cyto.a.22491), Hillmann et al., 2016 (doi: 10.3727/096368915X687822) and Schubert et al., 2018 (doi: 10.1002/cyto.a.23240).

The human MSC included in the current study were largely positive for CD90, CD105 and CD44, partly positive for CD29 and negative for CD14, CD34, CD45, CD79α and MHCII.
The equine MSC were largely positive for CD44, partly positive for CD90, CD29 and CD105, and negative or largely negative for CD14, CD34, CD45, CD79α and MHCII.

The S1 Supplementary Table shows the median percentages of positive cells as determined by flow cytometry:

	CD90	CD105	CD44	CD29	CD14	CD34	CD45	CD79α	MHCII
human	98%	80%	66%	13%	0%	0%	0%	0%	0%
equine	41%	23%	93%	28%	1%	0%	0%	0%	0%

The human and equine MSC were also capable of trilineage differentiation. Panel A of the S1 Supplementary Figures shows representative images after 3 days of adipogenic induction and 21 days of osteogenic or chondrogenic induction, and the respective stainings.

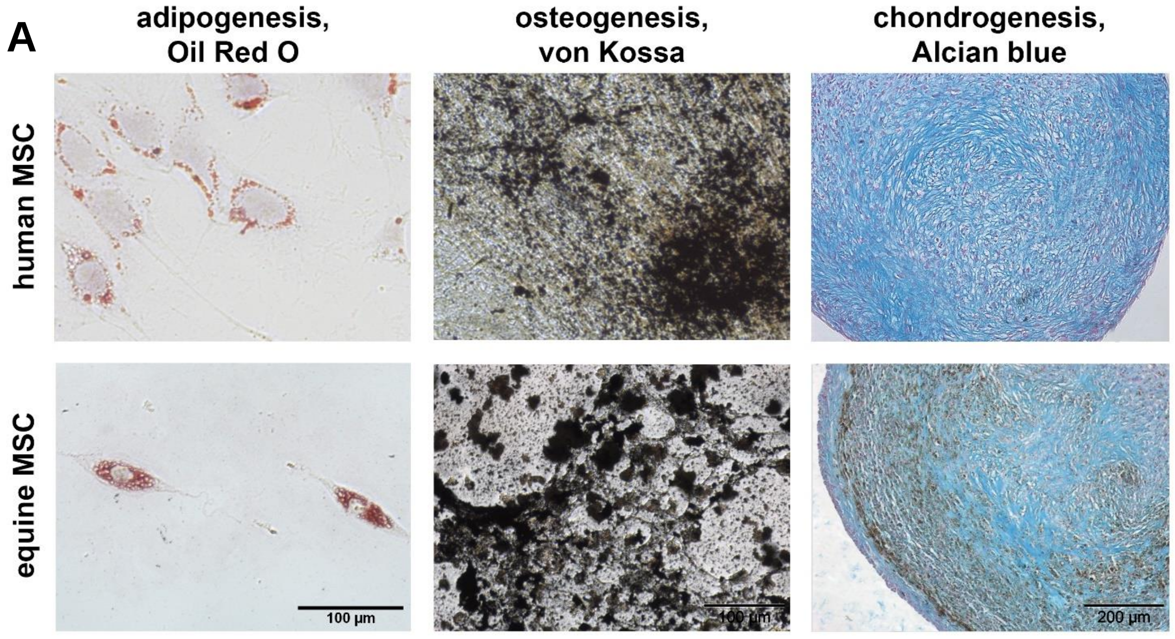

Fibroblast characterization:

Representative samples of the human scar tissue-derived fibroblasts were characterized by their gene expression after culture in standard conditions and as compared to commercially available normal human dermal fibroblasts (PromoCell, Heidelberg, Germany). Furthermore, trilineage differentiation potential of the scar tissue fibroblasts was evaluated, with prolonged incubation times as compared to standard MSC differentiation.

Panel B of the S1 Supplementary Figures illustrates the qualitative analysis of gene expression in the scar tissue fibroblasts (FB), which corresponded to the normal human dermal fibroblasts (NHDF) for all genes analyzed. These included genes encoding for the surface antigen CD90 (THY1), the proliferation-associated cyclin D1 (CCND1), and markers of trilineage differentiation (PPARG2, RUNX2, ACAN).

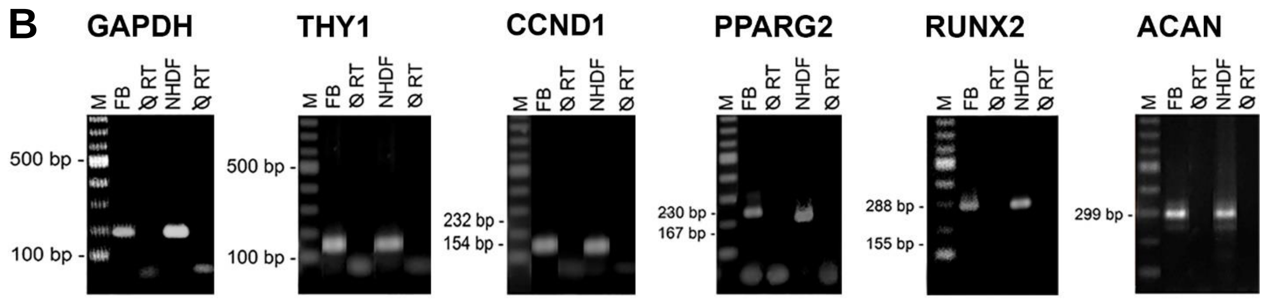

Panel C of the S1 Supplementary Figures shows the scar tissue fibroblasts after 6 weeks of adipogenic induction, 3 months of osteogenic induction and 4 weeks of chondrogenic induction, and the respective stainings. Note that part of the fibroblast population was capable of differentiation.

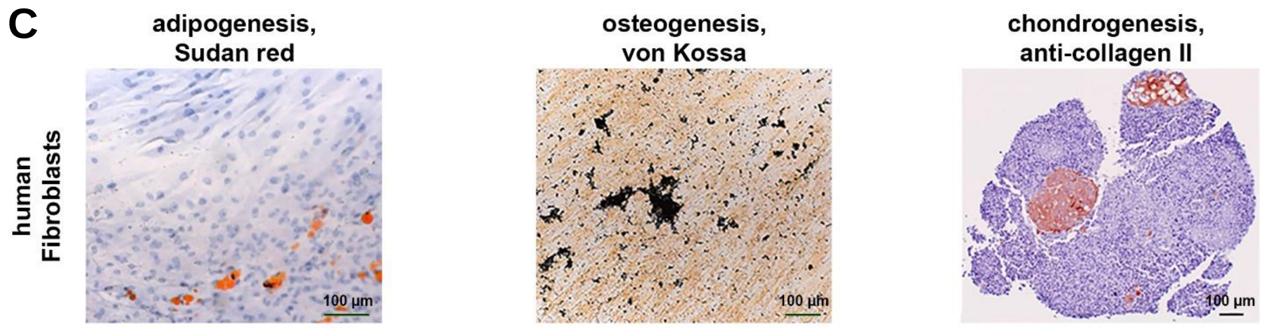

Supplement: Supplementary file 3 [file DataSheet1.PDF]
